# Simulation of Neural Signals’ Spectrum and Transmission from the Perspective of Impedance-Matching Effect of the Ranvier Node

**DOI:** 10.3390/bioengineering13080955

**Published:** 2026-08-21

**Authors:** Mingwei Song, Zhiming Xu, Jingjing Xu

**Affiliations:** 1 School of Integrated Circuits, Shandong University, Jinan 250101, China; 202300400168@mail.sdu.edu.cn; 2 Key Laboratory for the Physics and Chemistry of Nanodevices, School of Electronics, Peking University, Beijing 100871, China; 2501112236@stu.pku.edu.cn

**Keywords:** neural electromagnetic signals, Node of Ranvier, terahertz, waveguides, impedance matching

## Abstract

Addressing the current issues of the uncertain primary frequency bands and the difficulty in directly measuring the spectral characteristics of neural electromagnetic signals transmitted within nerve fibers, this study investigates the spectral and time-domain properties of the neural electromagnetic signals through finite element simulations, using the influence of the node of Ranvier on nerve conduction velocity as a breakthrough point, and develops a coaxial transmission line impedance transformation model. The research results demonstrate that the primary signals capable of effective transmission within nerve fibers may reside in the terahertz (THz) band. They also show that the node of Ranvier essentially functions by regulating its geometric length to achieve impedance matching between the node and the myelin sheath, thereby reaching the optimal efficiency for signal coupling. The above finding not only explains the mechanism underlying the non-monotonic variation in neural conduction velocity with the length of the node of Ranvier, but also provides the latest perspective for understanding the relationship and differences between transmembrane action potentials that accompany the generation of neural signals and information transmission carriers—electromagnetic signals. It may also provide a theoretical supplement to the understanding of signal frequency–timing issues in neural encoding.

## 1. Introduction

Neural electromagnetic signals, as the primary carriers of information transfer within biological entities, serve as the physical foundation for sensory processing, motor control, and high-level cognitive functions [1]. For decades, the classic Hodgkin–Huxley (H-H) model, based on ion diffusion and membrane currents, has been widely employed to explain the generation and conduction mechanisms of nerve impulses [2,3]. However, with the continued advancement of neuroscience research, several biological phenomena that are difficult to explain using traditional theories have gradually been revealed [4,5,6,7,8]. For example, classical cable theory uses “saltatory conduction” to explain the rapid transmission of signals in myelinated nerves. However, calculations by Bulai et al. showed that [4], owing to the screening effect of ionic solutions, the transverse electric field near ion channels extends only approximately 10 μm. In myelinated nerves, adjacent ion channels are separated by insulating myelin sheaths and may be several hundred micrometers or even several millimeters apart, far exceeding the effective range of the transverse electric field. Therefore, there is great controversy over whether ion currents can be established along such long internodal distances in myelinated nerves within the classical theoretical framework. Furthermore, Gonzalez-Perez et al. reported that two counter-propagating action potentials did not annihilate upon collision but continued to propagate independently [5], while Caputi et al. found that electric eels achieve submillisecond synchronization of electric-organ discharge over a length scale of approximately 1 m [6,7,8]; these phenomena cannot be fully explained by the conventional refractory-period mechanism or the stepwise propagation of action potentials. In recent years, to further elucidate the physical properties and transmission mechanisms of neural signals, researchers have proposed several alternative hypotheses, including mechanical electro-soliton theory [9,10], quantum information theory [11,12], quantum-confined superfluid theory [13,14], and the theory of neural soft-matter waveguides [15,16,17,18]. These hypotheses provide diverse perspectives for understanding neural signals and their transmission processes.

Among these, Xu et al. proposed the neural soft-matter waveguide theory in 2012 [15] which conceptualizes nerve fibers as natural waveguides composed of organic biological materials. The specific structure is defined as intracellular fluid–myelin sheath–extracellular fluid, where neural signals propagate through this waveguide in the form of electromagnetic waves [15,16,17,18]. In 2018, Micheli’s group in Italy utilized COMSOL software (COMSOL Multiphysics, a finite-element-based multiphysics simulation platform developed by COMSOL AB, Sweden) to simulate the generation and transmission of neural signals, demonstrating that these signals can couple into the waveguide structure for transmission [19]. From 2019 to 2024, various studies published in journals such as Adv. Funct. Mater. and Nano Res. investigated electromagnetic wave propagation in nerve fibers from multiple perspectives, including electromagnetic field distribution in axons, refractive index differences between components, attenuation rates, the possibility of photonic information transmission, and their evolutionary laws [20,21,22,23,24,25,26]. In 2025, Zhang et al. studied the transmission of mid-infrared neural signals in nerve fiber waveguides and used their findings to explain the underlying mechanisms of demyelinating diseases [27]. In 2026, Xu et al. verified the electromagnetic transmission mechanism and the influence of geometry in soft-matter waveguides through HFSS (High Frequency Structure Simulator, version 2022 R1, a three-dimensional full-wave electromagnetic simulation software developed by Ansys, Inc., USA) simulations and experiments on bullfrog nerves [28]. These studies have investigated the generation, coupling, confinement, and propagation of electromagnetic signals in neural structures from different theoretical and computational perspectives, thereby supporting the physical feasibility of the neural soft-matter waveguide hypothesis.

However, existing electromagnetic measurement technologies cannot meet direct measurement requirements for high-frequency neural electromagnetic signals due to their weak intensity and severe attenuation in neuronal culture medium. As of now, a consensus remains elusive within the academic community regarding the primary frequency bands of electromagnetic signals transmitted in nerve fibers, with controversy centered on low-frequency (kHz) [18,23,28,29] and high-frequency (THz) [19,20,21,22,24,25,26,27] bands. For instance, Zangari and Xiang et al. calculated that neural electromagnetic signals reside in the terahertz and infrared bands (10^11^ Hz to 10^13^ Hz) by equating sodium ion channels to biological nano-antenna arrays and transport channels, respectively [19,22]. Zhai et al., using electromagnetic mode solvers, found that signals ranging from 10^4^ Hz to 10^11^ Hz can transmit effectively within neural waveguides [23]. Conversely, Wang et al. discovered that the physical process following the opening of sodium ion channels excites electromagnetic waves at frequencies of 10^3^–10^6^ Hz and 10^7^–10^8^ Hz [29]. Scans of the S_21_ parameter further identified an optimal transmission band at 10^3^–10^6^ Hz [28]. Here, S_21_ denotes the forward transmission scattering parameter, defined as the ratio of the voltage-wave amplitude received at Port 2 to that incident at Port 1 under impedance-matched conditions. This discrepancy makes results from different research groups difficult to compare. Now, investigating the spectral characteristics of neural signals through indirect methods has become an indispensable research approach over time. For example, Xu et al. explored frequency transmission characteristics by constructing simulation models and performing spectral scans [28], while Kumar et al. inferred transmission bands by examining photon-coupling efficiency in nerve fibers [30]. Previous studies have shown a compelling characteristic of neural signal transmission: the conduction velocity of neural electromagnetic signals exhibits a non-monotonic correlation with the geometric length of the node of Ranvier. Specifically, when other structural factors—such as myelin thickness and axonal inner diameter—remain constant, the conduction velocity first increases and then decreases as the length of the node of Ranvier increases [31,32,33]. The optimal conduction velocity corresponds to a nodal length of approximately 1.5 μm to 2 μm [31]. This phenomenon may imply a specific response and modulation law of nerve fibers toward certain signal frequencies.

In this paper, the influence of the node of Ranvier length on neural signal transmission is taken as an entry point. Because this problem involves electromagnetic-wave propagation in a heterogeneous and multilayered nerve-fiber structure, finite-element analysis is particularly suitable [34]. Using this method, we constructed a nerve-fiber model comprising a node of Ranvier and the subsequent myelin segment. We then simulated signal transmission across different frequency bands and nodal lengths to infer the principal transmission bands and their characteristics.

## 2. Materials and Methods

### 2.1. Relationship Between the Length of the Node of Ranvier and Neural Signal Transmission Characteristics

**Figure 1:**
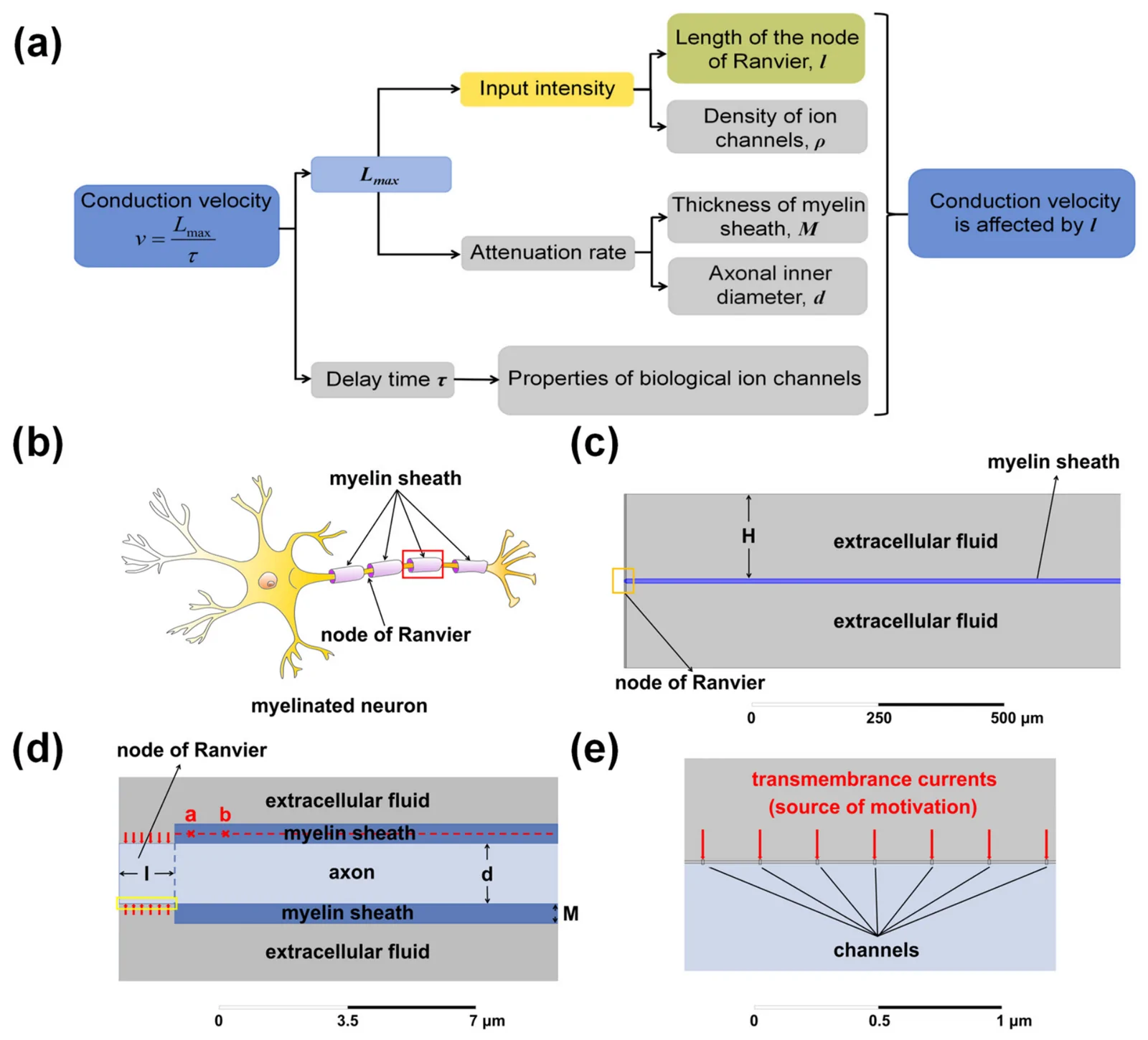
Factors influencing the signal conduction velocity of myelinated nerve fibers and a schematic of the simulation model. (**a**) Primary factors influencing the signal conduction velocity in myelinated nerve fibers, where gray boxes represent factors held constant in this study. (**b**) Schematic diagram of the myelinated nerve fiber structure, with the red box representing a periodic functional unit for signal generation and transmission. (**c**) Enlarged view of the red box region, where H represents the thickness of the extracellular fluid surrounding the nerve fiber. (**d**) Enlarged view of the orange box region; the red arrows represent the transmembrane current at the node of Ranvier, and the red dashed line represents the radial midline of the myelin sheath region. The electric field amplitude at point a is defined as the initial amplitude of the neural electromagnetic signal fed into the myelin sheath, with point a located 5 μm axially from the junction of the node of Ranvier and the myelin sheath. Point b is located 20 μm axially from the same junction. (**e**) Enlarged view of the yellow box region, showing the uniform distribution of sodium ion channels on the cell membrane.

In the present study, the term “neural signal” specifically refers to the electromagnetic wave transmitted through the nerve-fiber waveguide from one node of Ranvier to the next, rather than to the action potential. The latter is manifested as the temporal variation in transmembrane voltage at the node of Ranvier, whereas the former refers to the electromagnetic wave excited in the nodal region and coupled into the intracellular fluid–myelin sheath–extracellular fluid waveguide structure for propagation. Throughout this study, “signal amplitude” specifically denotes the magnitude (|**E**|) of the electric-field vector (**E**) of this electromagnetic wave, expressed in V/m. To investigate the influence of the node of Ranvier length on neural signal transmission characteristics, we selected the electric-field magnitude of the electromagnetic wave measured at point a as the primary observation metric in the simulations and hereafter refer to it as the “initial amplitude.” As shown in Figure 1d, point a is located 5 μm axially into the myelinated region from the junction between the node of Ranvier and the myelin sheath. The rationale is detailed below.

In myelinated nerve fibers, voltage-gated sodium (Na^+^) channels exhibit a highly regional distribution, primarily concentrated at the node of Ranvier, which serves as the critical site for the generation and conduction of action potentials. Simultaneously, the axon, the myelin sheath wrapping the axon, and the external extracellular fluid together construct a cylindrical soft-matter waveguide (intracellular fluid–myelin sheath–extracellular fluid), providing an efficient physical channel for the transmission of neural electromagnetic signals. Electromagnetic waves propagate at approximately 10^8^ m/s, far faster than the measured apparent nerve conduction velocity of 0.01–100 m/s [31,35]. This difference suggests that the nodal relay is the rate-limiting stage of conduction. The relay comprises sodium-channel opening, transmembrane-current generation, and excitation of a new neural electromagnetic signal. The time consumed by this process is typically referred to as the signal delay time, *τ*, which remains nearly constant within the same neuron [36]. Consequently, the measured apparent conduction velocity *v* is proportional to the average length between nodes of Ranvier (hereafter referred to as the average internodal length), *L_in_*, as shown in Equation (1); this is consistent with previous studies [36,37,38]. From an evolutionary perspective, organisms tend to maximize the average internodal length to reduce the number of nodes per unit length (i.e., the number of relays), thereby increasing the nerve conduction velocity and enhancing survivability. On this basis, we hypothesize that, in myelinated nerve fibers, the maximum transmission distance of a neural electromagnetic signal generated by a single excitation, *L_max_*, may be approximated by the average internodal length *L_in_*, yielding:

(1) $$v=\frac{L_{all}}{T_{all}}=\frac{N\cdot L_{in}}{N\cdot \tau }=\frac{L_{in}}{\tau }\approx \frac{L_{\max}}{\tau },$$

where *L_all_* and *T_all_* represent the total length of a nerve fiber and the total time for a neural signal to conduct along it, respectively, and *N* represents the number of nodes of Ranvier (which also corresponds to the number of myelin segments).

Figure 1a illustrates the primary factors influencing signal conduction velocity. Since the delay time *τ* remains nearly constant in the same neuron, the conduction velocity *v* is proportional to the maximum transmission distance *L_max_*. In turn, *L_max_* is affected by the initial amplitude of the neural electromagnetic signal fed into the myelin sheath and its transmission loss within the myelin (hereafter referred to as the attenuation rate). Specifically, the length of the node of Ranvier (*l*) and the ion channel density (*ρ*) thereon influence the initial amplitude, whereas the myelin thickness (*M*) and axonal inner diameter (*d*) determine the attenuation rate of the neural signal [28].

Within the framework adopted here, the length of the node of Ranvier *l* may affect *L_max_*—and thus alter the conduction velocity—by influencing the initial amplitude. When the myelin thickness *M* and axonal inner diameter *d* remain constant, the attenuation rate of the neural signal in the myelin sheath may be treated as approximately constant. In this case, *L_max_* and the conduction velocity *v* are expected to be positively correlated. Furthermore, when the ion channel density *ρ* is also held constant, the number of ion channels capable of generating neural electromagnetic signals at the node increases as the length of the node of Ranvier *l* increases. Logically, the initial amplitude of the neural signal should increase monotonically. However, previously reported experimental results show that the conduction velocity *v* follows a non-monotonic trend—first increasing and then decreasing—as the length of the node of Ranvier increases [31,32,33]. This contradicts the expected inference and suggests that, in addition to altering the number of ion channels, there must be other mechanisms by which the length of the node of Ranvier influences the initial amplitude.

### 2.2. Geometric Model of Myelinated Nerve Fibers

In this study, a periodic functional unit with signal generation and transmission capabilities—consisting of a single node of Ranvier and its subsequent myelin segment—was constructed using COMSOL Multiphysics, version 6.2 (COMSOL AB, Stockholm, Sweden), based on finite element analysis (FEA). The generation and transmission of neural signals were simulated using this model. Furthermore, frequency sweep analysis of the S_21_ parameters of the nerve fiber under excitation across different frequency bands was performed using Ansys HFSS, version 2022 R1 (ANSYS, Inc., Canonsburg, PA, USA).

The electromagnetic and geometric parameters used for modeling were based on previously reported values. As shown in Figure 1b–e, in the constructed myelinated axon model, the axonal inner diameter (*d*) is 3 μm, and the myelin thickness (*M*) is 1 μm [28]. The thickness of the cell membrane at the node of Ranvier is 10 nm. The myelinated portion, excluding the node of Ranvier, is 1 mm long, while the length of the node of Ranvier (*l*) was set within the range of 1.0–4.5 μm as required.

Since the principal frequency band for signal transmission in nerve fibers is not yet established, and given that electromagnetic waves at different frequencies display intrinsically different propagation behaviors in biological tissues (e.g., dielectric response, loss mechanisms, and wavelength scaling), which lead to entirely distinct transmission modalities, we have accordingly examined the parameter ranges pertinent to both low- and high-frequency regimes in this simulation. Additionally, when defining the parameter values for the model, we consulted published experimental data to ensure biological plausibility. Previous studies generally regard signals below 1 MHz, particularly those between 1 kHz and 1 MHz, as relatively low-frequency signals. Our preliminary simulations showed similar electromagnetic characteristics across this frequency range; therefore, 100 kHz was selected as a representative low-frequency excitation frequency. For the low-frequency (100 kHz) excitation source, the material inside the axon was set as a non-solid with an electric conductivity of 0.487 S/m, a relative magnetic permeability of 0.999991, and a relative permittivity of 136 to simulate the intracellular fluid environment [39,40,41]. The myelin material was set as a solid with an electric conductivity of 0 S/m, a relative magnetic permeability of 1, and a relative permittivity of 10.3 to simulate realistic biological myelin conditions [39,40]. Outside the myelin sheath, the entire nerve fiber model was encapsulated in a liquid material with a thickness of 97.5 μm (*H*), an electric conductivity of 1.454 S/m, a relative magnetic permeability of 0.999991, and a relative permittivity of 136, simulating the extracellular fluid environment [39,41,42,43,44]. As for the high-frequency part, given that some theoretical studies and indirect experimental evidence, from aspects such as photon signal detection, intrinsic vibrational modes, and nanoantenna radiation, have suggested the possible existence of THz signals in nerve fibers [19,20,22,45], this study thus uses this frequency band as the representative of the high-frequency excitation. For the high-frequency (terahertz) excitation source, the relative permittivity of the materials changes because various polarization mechanisms require a certain response time, and molecules cannot synchronize with the rapidly alternating electric field. According to existing research, the relative permittivities of the intracellular fluid, myelin, and extracellular fluid at high frequencies were adjusted to 1.69, 2, and 1.44, respectively [20,27,46].

In biological myelinated nerve fibers, transmembrane sodium (Na^+^) channels are concentrated at the node of Ranvier, which serves as the site for neural signal generation. As shown in Figure 1e, to simulate the function of sodium channels, rectangular lumped current ports (20 nm in length and 10 nm in width) were placed on the node of Ranvier as excitation sources, with the current flowing inward perpendicular to the cell membrane surface. Given the requirements for model complexity, the linear density of the current ports on one side was set to 5 μm^−1^, with the ports uniformly distributed along the node of Ranvier. A current amplitude of 1 pA was employed solely as a normalized input to excite electromagnetic waves at the corresponding frequencies. This value does not represent the actual amplitude of physiological signals.

### 2.3. Physics and Boundary Conditions

The “Electromagnetic Waves, Transient” (temw) module was selected for the simulation to observe the wave-like nature of the signals and their attenuation during propagation. The governing equations primarily utilized by the temw module are as follows:

(2) $$\nabla \times {\mu_{r}}^{-1}(\nabla \times A)+\mu_{0}\sigma \frac{\partial A}{\partial t}+\mu_{0}\frac{\partial }{\partial t}(\varepsilon_{0}\varepsilon_{r}\frac{\partial A}{\partial t})=0,$$

where *μ_r_* is the relative magnetic permeability of the material, *μ*_0_ is the vacuum magnetic permeability, ***A*** is the magnetic vector potential, *ε_r_* is the relative permittivity of the material, *ε*_0_ is the vacuum permittivity, and *σ* is the material electric conductivity. This equation is the time-dependent electromagnetic wave equation formulated in terms of the magnetic vector potential. It is derived directly from Maxwell’s equations, specifically the Ampère-Maxwell Law and Faraday’s Law of Induction. The first term represents the magnetic diffusion (the spatial propagation and distribution of the magnetic field); the second term indicates the induced electric currents (eddy currents) and heat dissipation; the third term shows the displacement current (polarization).

To avoid the influence of electromagnetic wave reflections at the boundaries and ensure the accuracy of the results, while also considering model complexity requirements, the extracellular fluid is approximated as an infinite open space. A Scattering Boundary Condition was therefore applied to its outer boundary. During the mesh generation process, the mesh size was controlled by the physics-controlled sequence.

## 3. Results and Discussion

### 3.1. Primary Signal Frequency Bands Transmitted in Nerve Fibers

To investigate the primary frequency bands of signals transmitted in nerve fibers, this study simulated the initial amplitude of neural electromagnetic signals (the electric-field amplitude at point a in Figure 1d) fed into the myelin sheath and their propagation within nerve fibers under four commonly reported excitation frequencies: 100 kHz, 10 THz, 20 THz, and 40 THz. Considering the general distribution range of the length of the node of Ranvier in biological axons [31,32,33], the simulated length of the node of Ranvier was set from 1 μm to 2.5 μm (or 4.5 μm for 10 THz), with step sizes of 0.25 μm.

**Figure 2:**
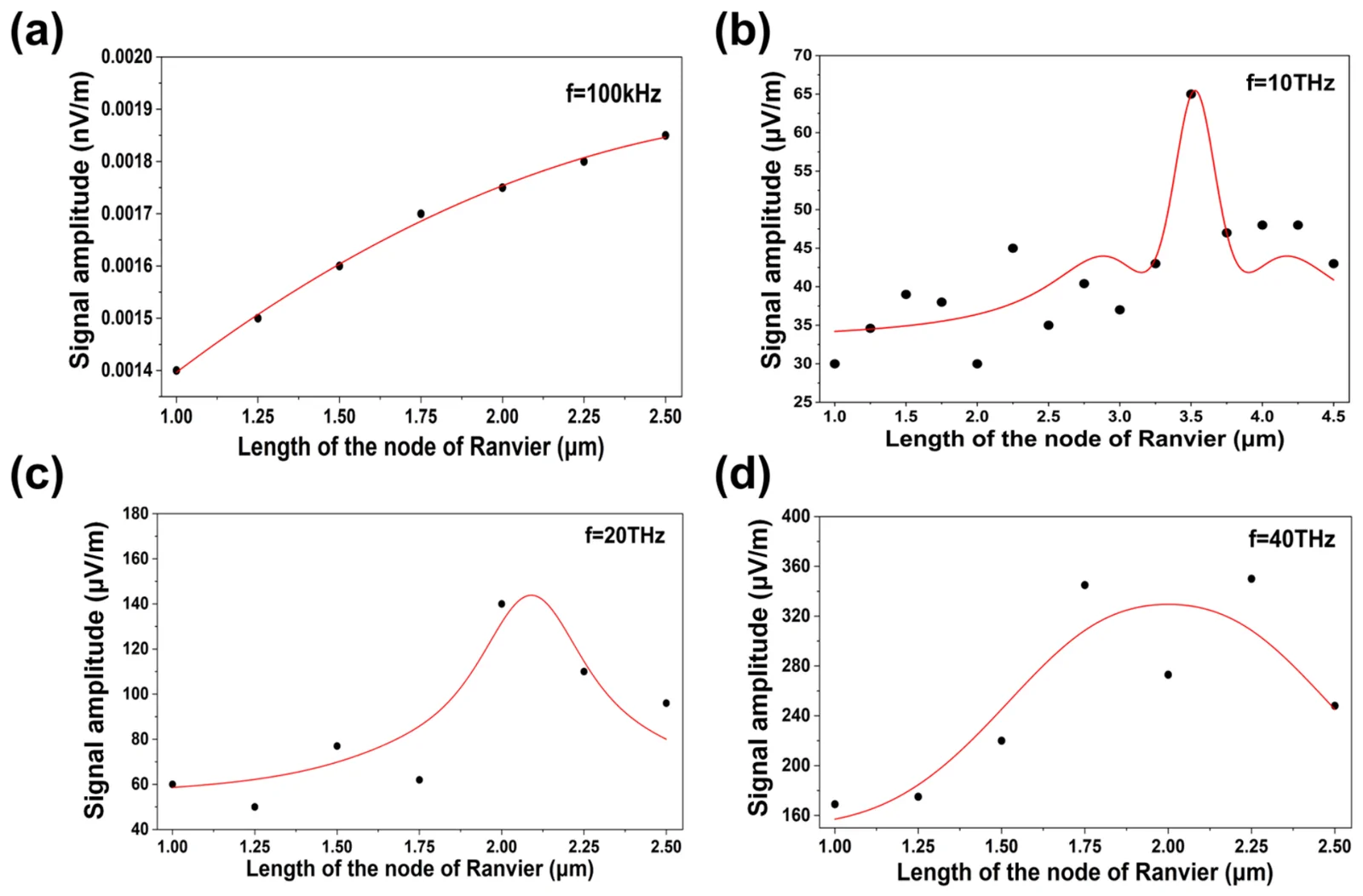
Relationship between the initial amplitude of the electromagnetic signal at the node of Ranvier and the length of the node of Ranvier as revealed by simulation results. (**a**) Relationship between electric field amplitude and the length of the node of Ranvier at an excitation frequency of 100 kHz. (**b**) Relationship between electric field amplitude and the length of the node of Ranvier at an excitation frequency of 10 THz. (**c**) Relationship between electric field amplitude and the length of the node of Ranvier at an excitation frequency of 20 THz. (**d**) Relationship between electric field amplitude and the length of the node of Ranvier at an excitation frequency of 40 THz. The black dots in the figures represent the raw data, and the red solid lines indicate the general trends. Specifically, the red solid lines in panels (**b**–**d**) were obtained by single-peak fitting based on the PsdVoigt2 function.

The simulation results presented in Figure 2 demonstrate that under low-frequency (100 kHz) excitation, the initial signal amplitude exhibits a monotonic positive correlation with the length of the node of Ranvier (Figure 2a). In contrast, under high-frequency excitation (10 THz, 20 THz, and 40 THz), the initial signal amplitude follows an overall trend of first increasing and then decreasing as the length of the node of Ranvier increases (Figure 2b–d), which is consistent with the literature reports [28,31,32,33]. As described by the equation above, these trends in initial signal amplitude may provide an indirect indication of corresponding changes in nerve conduction velocity. This observation suggests that the electromagnetic signals efficiently transmitted in nerve fibers may reside in the terahertz range. Notably, the length of the node of Ranvier corresponding to the peak initial signal amplitude under high-frequency excitation is closely related to the excitation frequency. For instance, at 10 THz, the initial amplitude reaches a maximum of 65 μV/m when the length of the node of Ranvier is 3.5 μm; at 20 THz, it peaks at 140 μV/m with a length of 2 μm; and at 40 THz, it reaches a maximum of 345 μV/m at a length of 1.75 μm. This suggests that the influence of the length of the node of Ranvier on neural signal conduction characteristics indeed reflects the response and modulation laws of nerve fibers toward signals of specific frequencies.

#### 3.1.1. Lossy Coaxial Cable Model for Low-Frequency Signals

Under low-frequency excitation at 100 kHz, variations in the length of the node of Ranvier do not trigger any significant signal modulation patterns. As shown in Figure 2a, the initial signal amplitude is positively correlated with the length of the node of Ranvier, which can be attributed to the increase in the number of sodium ion channels at the node. In fact, in a low-frequency environment, the axon can be modeled as a highly lossy coaxial cable structure [47], where the intracellular and extracellular fluids serve as the conductors on both sides, and the myelin sheath acts as the intermediate insulating dielectric layer. Under these conditions, the calculated skin depth is approximately 1–2 m, which is much larger than the axonal dimensions; thus, the myelin sheath fails to provide effective electromagnetic confinement. Furthermore, with a wavelength on the scale of kilometers, the phase variation in the signal along the structure is nearly zero. The electric field distribution is almost entirely determined by the charge distribution and remains independent of minor changes in the length of the node of Ranvier.

**Figure 3:**
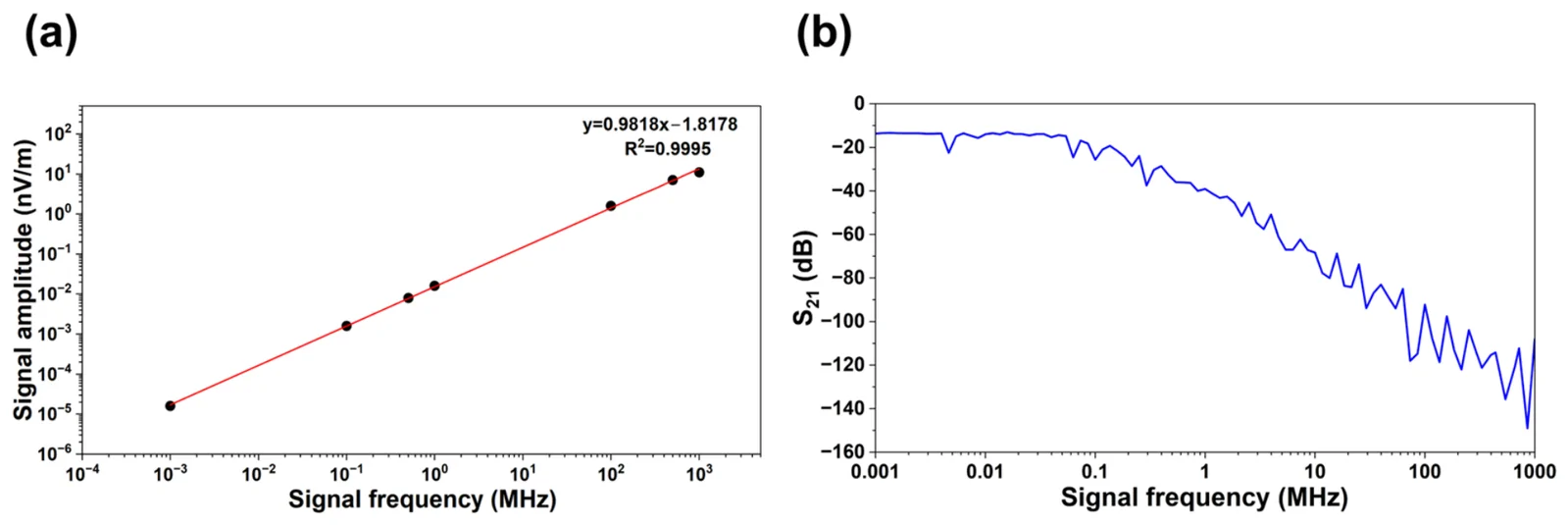
Relationship between the initial amplitude of the excited signal and the excitation frequency, and the S_21_ parameter within the myelin segment versus frequency, under low-frequency (1 kHz–1 GHz) excitation. (**a**) Relationship between the initial amplitude of the neural electromagnetic signal fed into the myelin sheath and the excitation source frequency with the length of the node of Ranvier fixed at 1.5 μm. The black dots represent the data points, and the red solid line represents the linear fit to the data. (**b**) S_21_ parameters for signal transmission within the myelin segment at different frequencies.

According to derivations from Maxwell’s equations, under quasi-static conditions, the initial amplitude of the excited electric field should be proportional to the frequency of the excitation source (See detailed calculation process in Section S1 in Supplementary Materials). The simulation results, as shown in Figure 3a, indicate that when the signal frequency is below 100 MHz, the initial amplitude of the excited electric field is strictly proportional to the signal frequency. Even when considering a frequency range from 1 kHz to 1 GHz, the electric field amplitude and signal frequency maintain an approximately linear relationship, with a linear fit R^2^ = 0.9995. This aligns with the inference of a proportional relationship between initial amplitude and signal frequency under the lossy coaxial cable model hypothesis. Meanwhile, at low frequencies, the low electric conductivity of the cellular fluid leads to severe Ohmic losses as electromagnetic waves propagate through the neural soft-matter waveguide (myelin segment), resulting in rapid signal attenuation. To quantify signal transmission within the myelinated segment, Port 1 and Port 2 were defined at the beginning and end of the segment, respectively. Specifically, Port 1 was located at the intersection of the central axis of the myelin sheath (the red dashed line in Figure 1d) and the boundary on the starting side, whereas Port 2 was located at the corresponding intersection with the boundary on the ending side. S_21_ represents the forward transmission coefficient from Port 1 to Port 2 through the myelinated segment. As shown in Figure 3b, the S21 parameter continuously decreases as the frequency increases. When the frequency exceeds 100 MHz, the S_21_ parameter drops below −100 dB, indicating extremely low energy transmission efficiency for low-frequency signals in subsequent myelin segments. Therefore, by combining the simulation results of the initial excited electric field amplitude (Figure 2a) and the signal transmission efficiency (Figure 3b), it can be concluded that low-frequency signals are likely not the primary frequency band for electromagnetic transmission within nerve fibers.

#### 3.1.2. Optical Fiber-like Model for High-Frequency Signals

When the excitation source is in the terahertz (THz) frequency range, as shown in Figure 2b–d, the initial amplitude of the neural electromagnetic signal fed into the myelin sheath first increases and then decreases with the length of the node of Ranvier. For instance, when the signal frequency is between 20 and 40 THz, the maximum signal amplitude occurs at a length of the node of Ranvier of 2–1.75 μm, which is consistent with electrophysiological experiments and simulation results showing maximum values around 1.5–2 μm [28,31]. Previous studies have indicated that under high-frequency excitation, the axon can be modeled as an optical fiber-like structure [19,30,48], where the myelin sheath acts as an intermediate high-refractive-index dielectric layer that effectively confines electromagnetic waves for high-efficiency transmission. At a signal frequency of 20 THz, the wavelength within the myelin is approximately 10.6 μm, which is on the same order of magnitude as the length of the node of Ranvier, meaning phase effects cannot be neglected.

**Figure 4:**
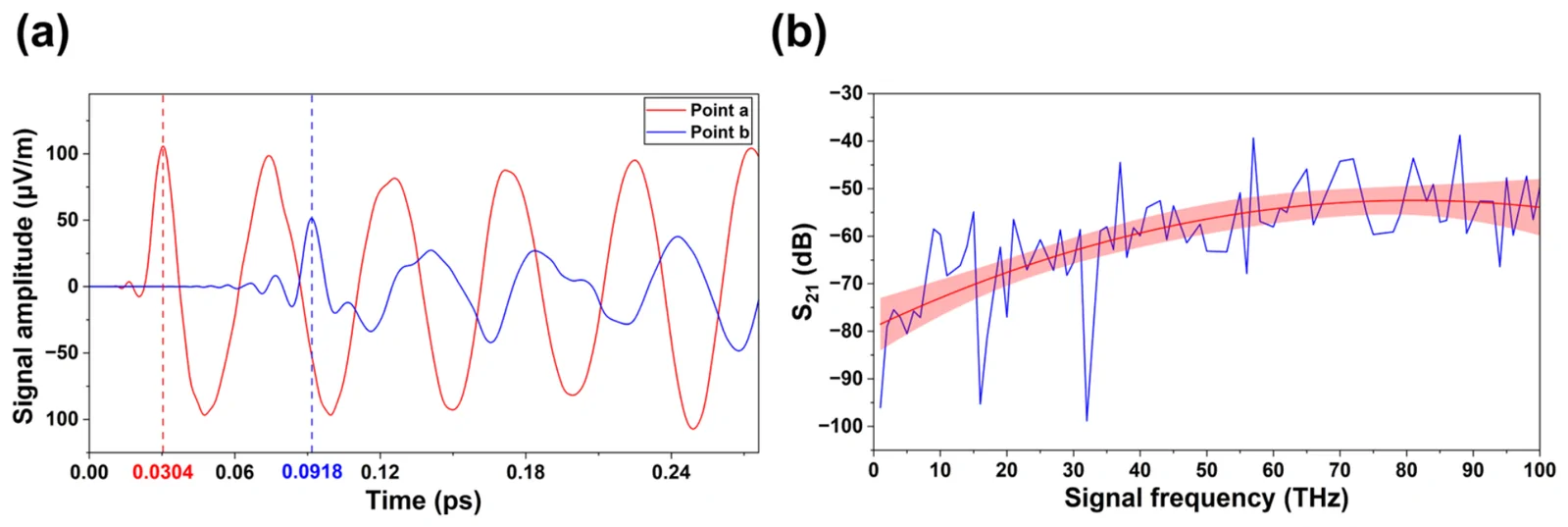
Time-domain signals within the myelin sheath and S_21_ parameters for different frequencies under high-frequency (1 THz−100 THz) excitation. (**a**) Time-domain signals at two observation points, a and b, on the myelin sheath during signal transmission simulation. The red and blue dashed lines, together with their corresponding time values, indicate the reference time points used to calculate the electromagnetic wave propagation velocity. (**b**) S_21_ parameters of the myelin segment under different excitation source frequencies, where the red solid line represents the fitted curve and the red shaded area represents the 95% confidence band.

Based on the optical fiber model, the propagation velocity of electromagnetic waves is given by $v_{1}=\frac{c}{\sqrt{\varepsilon_{r}\mu_{r}}}$, where c is the speed of light in vacuum, *ε_r_* is the relative permittivity of the medium, and *μ_r_* is the relative magnetic permeability (taken here as 1). By substituting the relative permittivities of the intracellular fluid (1.69), myelin (2), and extracellular fluid (1.44), the theoretical propagation velocity of electromagnetic waves in the myelin segment is calculated to be approximately 2.12 × 10^8^ m/s to 2.5 × 10^8^ m/s. Based on the time-domain simulation results of signal transmission (Figure 4a), the first distinct wave peak was taken as a marker at two points, a and b (Figure 1d), which are 15 μm apart along the axis. By dividing the distance between these two observation points by the time interval between the peaks, the electromagnetic wave propagation velocity was calculated to be 2.44 × 10^8^ m/s, aligning with theoretical predictions. At high frequencies, since the myelin acts as a low-loss dielectric, the primary loss mechanism for the electromagnetic field shifts from severe Ohmic loss to radiation loss, resulting in slower signal attenuation. As shown in Figure 4b, the S_21_ parameter of the myelin segment generally increases with frequency at high frequencies, reaching a maximum of approximately −40 dB. This indicates that high-frequency signals (THz) exhibit higher energy transmission efficiency in subsequent myelin segments. Therefore, combining the simulation results of the initial excited electric field amplitude (Figure 2b,c) and the signal transmission efficiency (Figure 4b), high-frequency signals are more likely to be the primary frequency band for electromagnetic transmission within nerve fibers.

### 3.2. Signal Modulation Mechanism: The Node of Ranvier as an Impedance Transformer Affecting Coupling Efficiency

We utilize transmission line theory to explain the modulation of high-frequency signals by the length of the node of Ranvier. The core logic is that the non-uniform transmission line structure—comprising the node of Ranvier and the subsequent myelin segment—exhibits a significant impedance mismatch at the interface. This interface effect directly determines the initial amplitude of the neural signal (the electric-field amplitude at point a in Figure 1d) fed into the myelin segment, thereby influencing the effective transmission distance of the neural signals from the source. Although water and biological fluids exhibit strong absorption in the terahertz band [49,50,51], the myelin sheath has been demonstrated to act as an effective low-loss waveguide that tightly confines the majority of electromagnetic energy [20]. Given the micro-scale length of the node of Ranvier (1–2 μm), such fluid-induced attenuation does not qualitatively alter the impedance discontinuity at the interface. Therefore, to simplify calculations and highlight the physical essence of this structural boundary, a lossless transmission line model is adopted here for estimation.

**Figure 5:**
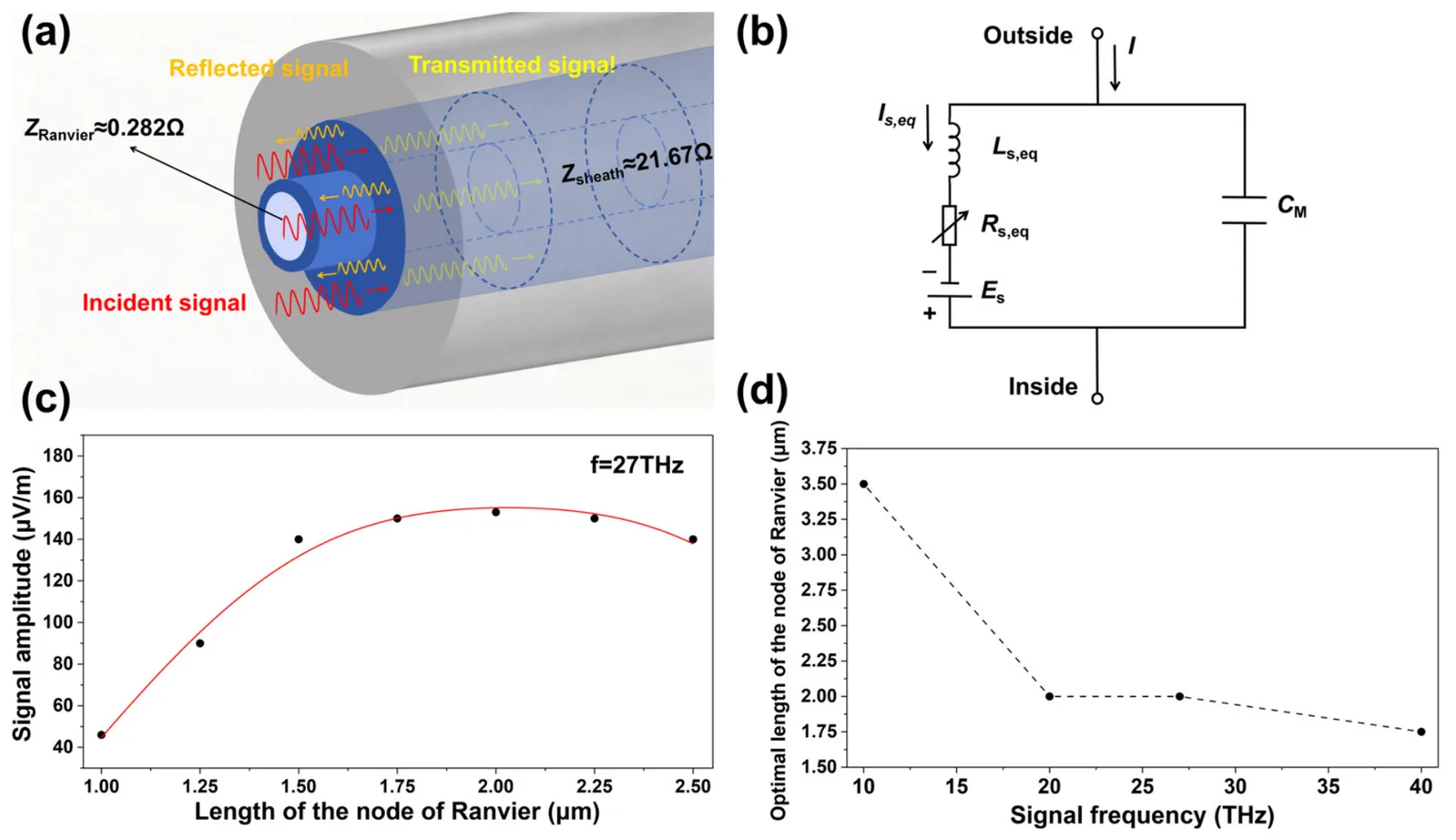
Schematic, circuit model, and simulation validation of the node of Ranvier as an impedance transformer. (**a**) Schematic of the incidence, transmission, and reflection of neural electromagnetic signals at the interface between the node of Ranvier and the myelin segment. The gray region represents the extracellular fluid, the dark blue region represents the myelin sheath or nodal membrane, and the light blue region represents the intracellular fluid. (**b**) Equivalent RLC circuit at the node of Ranvier. The arrows indicate the directions of the corresponding currents. (**c**) Relationship between the initial amplitude of the neural signal and the length of the node of Ranvier at an excitation frequency of 27 THz, where the black dots represent the simulated data and the red solid line indicates the fitted trend. (**d**) Lengths of the node of Ranvier corresponding to the maximum signal coupling efficiency at different excitation frequencies, where the black dots represent the simulated values and the gray dashed line connects the data points to indicate the trend.

As shown in Figure 5a, the axonal structure can be modeled as a coaxial transmission line consisting of intracellular fluid (conductor), myelin sheath (dielectric layer), and extracellular fluid (conductor). According to transmission line theory calculations, an impedance discontinuity exists between the characteristic impedance of the node of Ranvier segment ($Z_{\mathrm{Ranvier}}\approx 0.282\;\Omega$) and that of the subsequent myelin segment ($Z_{\mathrm{sheath}}\approx 21.67\;\Omega$), leading to a partial reflection of the neural electromagnetic signal energy upon reaching the junction (See detailed calculation process in Section S2 in Supplementary Materials).

To maximize transmission efficiency for high-frequency signals, impedance matching should be achieved as closely as possible. The node of Ranvier likely functions as an impedance transformer that regulates coupling efficiency, with its length directly determining the effectiveness of the matching. It should be emphasized that this modulation is not achieved by altering the attenuation coefficient of the subsequent myelin segment (which follows an optical fiber-like model rather than a transmission line model at high frequencies), but rather by modifying the interfacial transmission coefficient. As impedance matching improves, the effective power fed into the myelin segment increases, resulting in higher initial signal energy and, consequently, a longer effective transmission distance.

From the perspective of minimizing signal reflection, the transmission efficiency is ideally maximized when the length of the node of Ranvier equals one-quarter of the wavelength (acting as a quarter-wavelength impedance transformer). For instance, at a signal frequency of 20 THz, the wavelength is approximately 10.6 μm, yielding a theoretical optimal node length of 2.65 μm. This value is relatively close to the ~2 μm observed in electrophysiological experiments; the discrepancy may stem from factors such as the lack of strict synchronization in sodium channel activation and the neglected inductive effects.

To maximize energy transfer efficiency, the output impedance of the signal source must be the complex conjugate of the input impedance of the subsequent transmission line (the myelin segment). The input impedance formula for the transmission line is given by:

(3) $$Z_{\mathrm{in}}=Z_{\mathrm{Ranvier}}\frac{Z_{\mathrm{sheath}}+jZ_{\mathrm{Ranvier}}\tan(\beta l)}{Z_{\mathrm{Ranvier}}+jZ_{\mathrm{sheath}}\tan(\beta l)},$$

where *Z_in_* is the input impedance of the transmission line, *l* is the length of the node of Ranvier, and β is the phase constant derived from the wavelength. Calculations show that for a node length of 2 μm, the input impedance of the subsequent myelin structure is approximately 0.0043 − j0.115 Ω. For maximum energy transfer, the output impedance of the signal source should be 0.0043 + j0.115 Ω, indicating that the signal source would need to exhibit a weak effective inductive behavior. This condition can be equivalently represented by an RLC circuit, as illustrated in Figure 5b. Since an LC resonant circuit possesses frequency-selective properties, we hypothesize that the neural signal frequency capable of efficient coupling into the myelin must be close to the resonant frequency of this circuit. For simplicity in estimation, we adopt the lumped-element approximation here, while noting that at 20 THz the ratio *l*/*λ* ≈ 0.17, in fact, slightly exceeds the usual lumped-element validity boundary (*l*/*λ* < 0.1). The following values should therefore be viewed as rough estimates. This rough estimate is intended primarily to guide the choice of excitation frequency for the subsequent simulations, rather than to serve as an exact prediction.

Using the aforementioned parameters, the membrane capacitance at the node of Ranvier is calculated to be *C_membrane_* = 33.4 fF, and the equivalent inductance of the signal source is *L_s_*_,*eq*_ = 0.915 fH (see detailed calculation process in Section S3 in Supplementary Materials). Substituting values of *L_s_*_,*eq*_ and *C_membrane_* into the resonant frequency formula $f_{0}=\frac{1}{2\pi \sqrt{\mathrm{LC}}}$ for a parallel LC circuit yields a resonant frequency *f*_0_ = 28.8 THz. Considering that the thin cell membrane at the node cannot completely confine electromagnetic waves, a more accurate equivalent relative permittivity should be a weighted average of the intracellular fluid (1.69), myelin (2), and extracellular fluid (1.44). Adopting a conservative value of 1.7 (close to the intracellular fluid), the corrected resonant frequency is approximately 26.6 THz. Figure 5c illustrates the relationship between the initial amplitude of the neural signal fed into the myelin and the length of the node of Ranvier using 27 THz as the excitation frequency. The signal reaches a maximum when the node length is approximately 2 μm, and remains relatively stable for node lengths between 1.75 μm and 2.25 μm. The agreement between the estimated resonant frequency (~26.6 THz) and the simulation result—which shows a clear peak at *l* ≈ 2 μm under 27 THz excitation—supports the validity of our impedance matching hypothesis. Furthermore, the initial amplitude remains relatively stable when the length of the node of Ranvier ranges between 1.75 μm and 2.25 μm, demonstrating the robustness of the node of Ranvier as an impedance matcher.

Based on this analysis, as the signal frequency increases and the wavelength shortens, the node length corresponding to maximum coupling efficiency should also decrease. As shown in Figure 5d, our simulation results for 10 THz, 20 THz, 27 THz, and 40 THz signals confirm this trend, with their respective optimal lengths of the node of Ranvier being 3.5 μm, 2 μm, and 1.75 μm.

In summary, the node of Ranvier serves not only as the generation site for neural electromagnetic signals but also essentially functions as an impedance transformer that regulates the coupling of these signals into the subsequent myelin transmission line. Nodal length affects high-frequency signals through two mechanisms. First, a longer node contains more sodium channels and may increase the source amplitude. Second, nodal length alters impedance matching and therefore changes coupling efficiency. Coupling is highest at the optimal nodal length. When the node of Ranvier is at its optimal length, the signal coupling efficiency is maximized. As the length continues to increase beyond this point, the impedance becomes progressively mismatched, causing a decline in coupling efficiency. Consequently, even with an increased number of sodium ion channels, the transmitted signal amplitude fails to show significant gains and may even weaken. From an evolutionary perspective, the optimal length of 1.5–2 μm observed in electrophysiological experiments may represent the ideal balance between signal fidelity and energy transmission efficiency.

### 3.3. Time-Domain Characteristics

**Figure 6:**
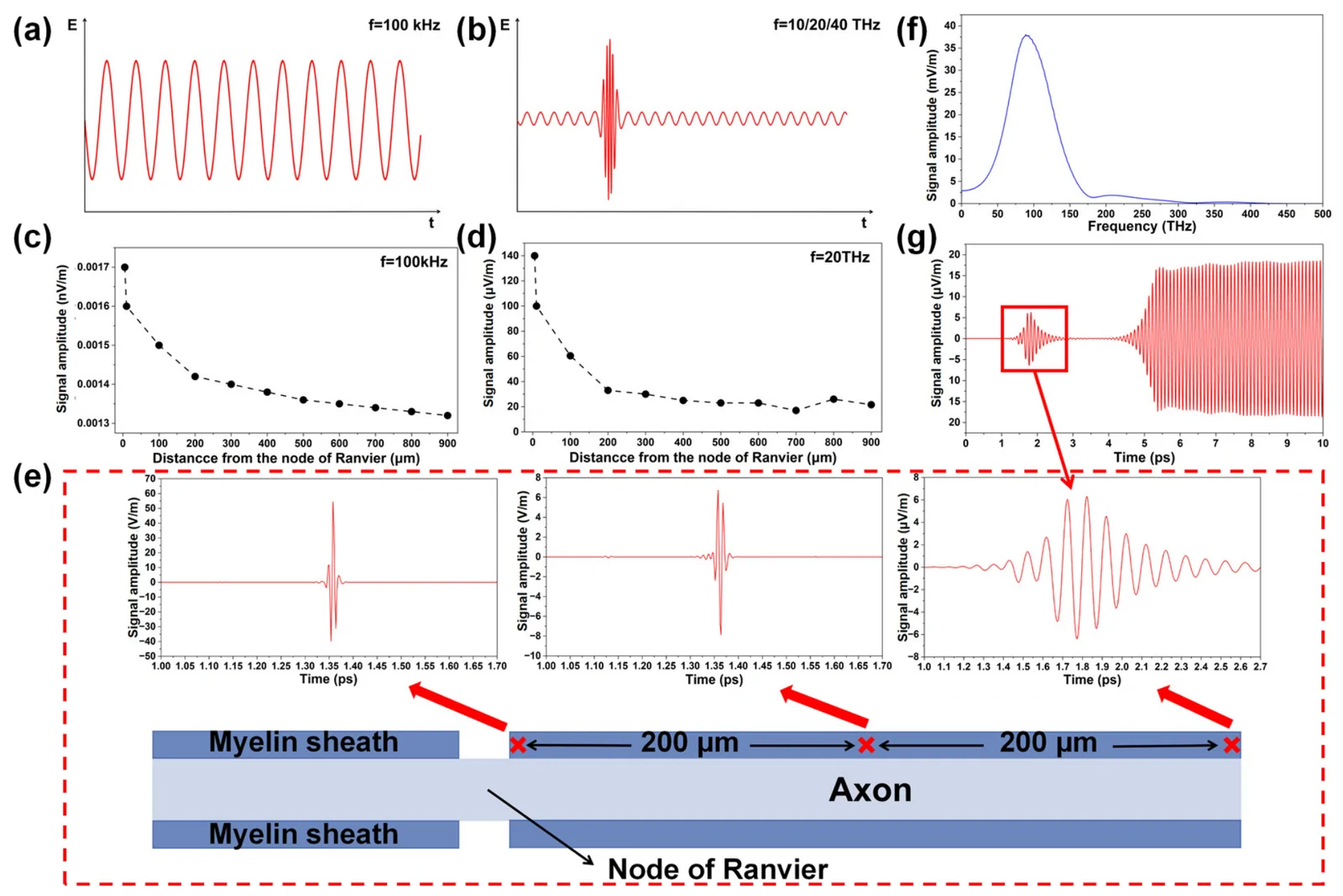
Schematic, simulation results, and time-frequency domain characteristics of signals in the myelin sheath under different frequency excitations. (**a**) When the excitation source is 100 kHz, the signal in the myelin sheath is a 100 kHz sine wave. (**b**) When the excitation source is in the terahertz range, the signal in the myelin sheath is a time-domain superposition of a fundamental sine wave at the source frequency and a high-amplitude pulse at a higher frequency. (**c**) Attenuation of the 100 kHz fundamental signal in the myelin sheath with distance (note: the vertical axis unit is nV/m). (**d**) Attenuation of the 20 THz fundamental signal in the myelin sheath with distance (note: the vertical axis unit is μV/m). (**e**) Time-domain waveforms of the neural signal after propagating different distances along the radial midline of the myelin sheath. The red dashed box indicates Panel (**e**). (The direction of transmission is from left to right.) (**f**) Frequency-domain analysis results of the time-domain signal on the left in Panel (**e**). (**g**) Dominance of the fundamental frequency signal after the neural signal has propagated 400 μm along the radial midline of the myelin sheath.

As illustrated in Figure 6a,b, time-domain simulation results of electromagnetic signal conduction in nerve fibers reveal distinct behaviors across frequency ranges. For low-frequency excitation (below 10 GHz), the signal propagating within the myelin sheath consists solely of a sine wave identical to the source frequency. In contrast, for high-frequency excitation in the terahertz (THz) range, the signal manifests as a superposition of a sine wave at the source frequency and a high-amplitude pulse. This indicates that the primary signal transmitted in nerves is the fundamental frequency signal matching the source, which exhibits exceptional spatial coherence and low propagation loss across different positions within the myelin sheath.

Interestingly, during the initial stage of high-frequency excitation, the time-domain waveform features a pulse with an amplitude significantly larger than the fundamental component. This pulse possesses two fundamental characteristics: (1) an extremely short duration, much smaller than the period of the fundamental signal, and (2) extremely rapid attenuation compared to the fundamental signal, decaying to approximately 10% of its initial amplitude after 200 μm (Figure 6e). According to the principles of Fourier transform, the spectrum of this pulse appears as a broad continuous spectrum centered at frequencies higher than the fundamental (Figure 6f).

Through modification of simulation conditions, we analyzed the potential causes of this anomalous transient pulse in the myelin sheath under high-frequency excitation. First, to simulate realistic physiological conditions, the outer boundaries were set as scattering boundary conditions, thereby excluding the influence of reflected waves from finite boundaries. Second, no pulses were observed in regions where the electromagnetic signal had not yet physically arrived, even if the timing coincided with the pulse’s appearance in the time domain; this confirms that the pulse is not a numerical artifact caused by calculation steps or mesh discretization. We further considered whether the pulse might result from the interference of signals excited by Na^+^ channels at different positions along the node of Ranvier. To test this, we modeled signal transmission with only a single ion channel at the node and still observed the same pulse, albeit with a slightly reduced amplitude, excluding the possibility of signal superposition from multiple sources. Given the significant thickness difference between the node of Ranvier and the subsequent myelin, we also investigated whether interface reflections were responsible. When the thickness of the subsequent myelin structure was set equal to that of the nodal cell membrane (effectively transforming the myelinated fiber into an unmyelinated one), the same pulse still appeared in the membrane, ruling out impedance mismatch-induced reflections as the cause.

Having excluded these possibilities, and based on waveguide mode analysis, this study posits that the physical essence of this anomalous pulse is the transient superposition of higher-order modes within the coaxial waveguide structure. On one hand, the abrupt change during the onset of the excitation source generates a broad spectrum centered around the fundamental frequency rather than a single-frequency initial spectrum. On the other hand, due to the high frequency (THz) of the excitation source, both the fundamental mode and numerous higher-order modes with higher cutoff frequencies are coupled into the nerve fiber. In waveguide theory, the superposition of electromagnetic field modes can be expressed as [52]:

(4) $$E(z,t)=\sum A_{n}e^{-\alpha_{n}z}e^{j(\omega t-\beta_{n}z)},$$

where *E* is the instantaneous electric field amplitude, *z* is the propagation distance, *t* is time, *A_n_* is the amplitude of the n-th component, *α_n_* is the attenuation constant of the n-th component, and *β_n_* is the phase constant. As per Equation (4), within short distances near the excitation source, the phases of the higher-order modes are close to those of the fundamental mode. Their amplitudes undergo coherent spatial superposition, forming a transient spike in the time domain that far exceeds the steady-state amplitude. However, since the attenuation constants *α_n_* of higher-order modes are significantly larger, these modes disappear rapidly as the propagation distance *z* increases due to the exponential decay factor and structural dispersion. The waveform eventually returns to a steady state dominated by the fundamental mode. Figure 6g displays the time-domain results after propagating 400 μm along the radial midline of the myelin sheath, showing that the pulse amplitude formed by higher-order mode superposition has decayed to a level far below that of the fundamental mode. These results explain the transient pulse as a superposition of higher-order modes and describe how the waveguide modes evolve during signal initiation.

## 4. Discussion

### 4.1. Spectral Characteristics of Neural Electromagnetic Signals

In summary, the aforementioned simulation and analytical results suggest that, during neural signal conduction, high-frequency neural signals may serve as the primary carriers of information. Within this framework, the myelin sheath may act as the main pathway for high-frequency signal propagation, while the node of Ranvier may function as an impedance matcher. By varying its length, the node may influence the coupling efficiency of neural signals, thereby affecting the apparent conduction velocity. Specifically, when a neural electromagnetic signal reaches a node of Ranvier, the voltage-gated sodium channels are activated. Consistent with the hypothesis adopted in this work, we consider that a high-frequency electromagnetic component on the order of 10 THz may be present at the node. For this signal to be efficiently coupled into the subsequent transmission line (the myelin segment), impedance matching would need to be satisfied. Through its specific length, the node of Ranvier may regulate this matching effect, thereby modulating the coupling efficiency of these signals into the myelin segment at specific frequencies. At high frequencies, the relative permittivity of the myelin sheath is greater than that of the intracellular and extracellular fluids on either side. This gradient forms a natural optical-fiber-like structure that may facilitate the confinement and relatively low-loss propagation of high-frequency electromagnetic waves. Collectively, these findings provide a coherent physical interpretation of how nodal geometry and the dielectric structure of myelinated nerve fibers may jointly regulate the coupling and transmission of high-frequency electromagnetic signals.

### 4.2. Dependence of the Optimal Nodal Length on Axonal Geometry

The dependence of the optimal nodal length, *l_opt_* (defined as the length of the node of Ranvier that maximizes conduction velocity), on axonal geometry provides another means of testing the impedance matching interpretation proposed in this study. According to this interpretation, *l_opt_* ≈ *λ*/*4*. Therefore, when the signal frequency, dielectric properties, and excitation conditions remain unchanged, *l_opt_* should be determined primarily by the signal wavelength rather than by the axonal inner diameter *d* or myelin thickness *M*. By contrast, in conventional cable models, the optimal nodal length is affected by membrane capacitance and axial resistance and should therefore vary with both *d* and *M*.

To test this prediction, we performed additional simulations at 20 THz while varying *d* and *M* separately. The baseline geometry was defined by d = 3 μm and M = 1 μm. Two additional geometries were considered: d = 5 μm with M = 1 μm, and M = 0.5 μm with d = 3 μm. For each geometry, the nodal length was varied from 1.0 to 2.5 μm in increments of 0.25 μm, while all other structural parameters, dielectric properties, and excitation conditions were held constant. We found that although changes in *d* or *M* affected the absolute electric-field strength, its dependence on nodal length was broadly similar among the three geometries (see Table S2 in the Supplementary Materials for detailed data). In all three cases, the largest sampled electric-field strength occurred at a nodal length of 2 μm. The pairwise Pearson correlation coefficients ranged from 0.861 to 0.912, and the corresponding Spearman rank correlation coefficients ranged from 0.865 to 0.955, indicating highly consistent trends among the three datasets.

Therefore, within the parameter ranges and sampling resolution examined in this study, the predicted optimal nodal length showed no appreciable dependence on either *d* or *M*. A direct quantitative comparison is difficult because cable models do not provide a generally applicable relationship among *l_opt_*, *d*, and *M*. Their predictions also depend on the selected internodal geometry, ion-channel density, membrane capacitance, axoplasmic resistance, and periaxonal geometry. We also reviewed the available morphometric evidence relevant to this prediction. Arancibia-Cárcamo et al. reported no significant dependence of nodal length on axon diameter in adult rat optic nerve and cortical axons [31], which is qualitatively consistent with the present results. However, the physiological nodal length does not necessarily correspond to the length that maximizes conduction velocity. Further comparisons using morphometric and electrophysiological data obtained under matched geometrical conditions are needed to test this prediction directly.

### 4.3. Limitations

Several limitations of the present study should be acknowledged. First, the empirical evidence for endogenous terahertz signals in nerve fibers remains limited. To date, neither direct spectrally resolved measurements nor independently reproducible experiments have confirmed the endogenous generation, absolute amplitude, or spectral distribution of terahertz signals during neural activity. The Ag^+^ photoreduction experiment reported by Zangari et al. [45] indicated neural-activity-associated photon emission at the nodes of Ranvier; however, the experiment lacked the spectral resolution required to determine the specific frequencies of these photons. Similarly, several studies have investigated the responses of neural tissues to externally applied terahertz radiation, but they have not demonstrated the generation of comparable endogenous signals. Therefore, the present study addresses a conditional question: assuming that high-frequency electromagnetic waves exist in nerve fibers, are high- or low-frequency signals are more likely to constitute the dominant frequency band for propagation? Accordingly, our findings should not be interpreted as experimental confirmation of either the endogenous generation or the spectral characteristics of a neural terahertz carrier.

Second, the current ports used in the simulations are normalized excitation sources employed solely to compare the relative coupling and propagation characteristics of signals at different frequencies. They do not represent the actual physiological amplitude of endogenous signals. Consequently, the calculated electric-field amplitudes cannot currently be used to predict the absolute power of terahertz signals under physiological conditions. In addition, the source impedance and inductance adopted in the equivalent-circuit analysis are effective parameters obtained through a conditional inverse construction based on the assumption of conjugate matching. The agreement between the resulting characteristic frequency and the full-wave simulation results demonstrates the internal consistency of the model, but does not establish that a specific biological structure possesses the predicted physical inductance or constitutes the actual terahertz source.

Finally, the present model is simplified and does not account for the effects of temperature on the electromagnetic properties of the materials or on signal generation, coupling, and propagation. It also does not incorporate the temperature-dependent nodal delay, *τ*(*T*). Therefore, the model cannot independently predict the experimentally observed temperature coefficient of conduction velocity. Future studies will focus on developing micro- and nanoscale local terahertz emission and reception structures integrated with electrophysiological measurements. Such experiments will be essential for determining the spectrum and absolute power of endogenous high-frequency signals, quantifying their coupling and transmission losses, and evaluating their relationship with neural conduction under physiologically relevant and temperature-dependent conditions.

## 5. Conclusions

This study investigated the spectral and time-domain properties of signals transmitted in nerve fibers by examining how nodal length affects signal transmission. We provide a possible physical explanation for the modulation effect of the length of the node of Ranvier on these signals. Our results suggest that the primary signals capable of effective transmission within nerve fibers may reside in the terahertz (THz) band. More importantly, the trend of neural conduction velocity varying with the length of the node of Ranvier—obtained through high-frequency signal generation and transmission simulations—is broadly consistent with existing electrophysiological experiments and computational results. This provides further theoretical support for the possibility that high-frequency electromagnetic signals may serve as carriers of neural information. Furthermore, this paper develops a coaxial transmission line impedance transformation model to provide a possible explanation for the modulation of neural signal frequency and conduction characteristics by the node. Specifically, within the framework adopted in this study, nodal length may regulate impedance matching between the node and the subsequent myelin sheath, thereby influencing the efficiency of signal coupling into the myelin segment. This research may contribute to understanding the possible physical mechanisms underlying the generation and transmission of neural electromagnetic signals and provide a complementary perspective on signal frequency–timing relationships in neural encoding.

## Data Availability

Data is contained within the article or Supplementary Materials.

## Supplementary Materials

The following supporting information can be downloaded at: https://www.mdpi.com/article/10.3390/bioengineering13080955/s1, Section S1: Calculation of the relationship between electric field intensity and signal frequency; Section S2: Calculation of characteristic impedance of different structural segments; Section S3: Calculation of membrane capacitance and equivalent inductance at the node of Ranvier; Table S1: Verification of the membrane capacitance and equivalent inductance calculations; Table S2: Simulated initial signal amplitude as a function of nodal length under different axonal geometries at 20 THz.
