# Supplementary material for: Simulation of Neural Signals’ Spectrum and Transmission from the Perspective of Impedance-Matching Effect of the Ranvier Node
Source: Bioengineering (Basel). 2026 Aug 21;13(8):955. doi: 10.3390/bioengineering13080955 (PMC13509889; doi:10.3390/bioengineering13080955)
Supplement: Supplementary file 1 [file bioengineering-13-00955-s001.zip › bioengineering-4449810-supplementary.pdf]

Supplementary Material

Simulation Study on Neural Signals' Spectrum and Transmission Characteristics from the Perspective of the Impedance Matching Effect of the Node of Ranvier

Mingwei Song[1], Zhiming Xu[2] and Jingjing Xu[1, *]

1 School of Integrated Circuits, Shandong University, Jinan 250100, China

2 Key Laboratory for the Physics and Chemistry of Nanodevices, School of Electronics, Peking University, Beijing 100871, China
* Corresponding author: xujj@sdu.edu.cn

Description: This material includes the detailed process of the calculation results that were briefly mentioned in the main text of the article.

CONTENTS

Section S1. Calculation of the relationship between electric field intensity and signal frequency
Section S2. Calculation of characteristic impedance of different structural segments
Section S3. Calculation of membrane capacitance and equivalent inductance at the node of Ranvier
Table S1. Verification of the membrane capacitance and equivalent inductance calculations
Table S2. Simulated initial signal amplitude as a function of nodal length under different axonal geometries at 20 THz

Section S1. Calculation of the relationship between electric field intensity and signal frequency

This calculation is performed under quasi-static conditions and is intended only for low frequencies (e.g., below 1 GHz), where the displacement current can be neglected. It is used to interpret the low-frequency simulation results in Section 3.1 and is not applied to the THz simulations. Under quasi-static conditions, the displacement current term in Maxwell's equations can be neglected, and the integral form of the Maxwell-Faraday equation can be written as:

$$\oint_C \mathbf{E} \cdot d\mathbf{l} = -\frac{d\Phi_B}{dt} \quad \text{(S1)}$$

where E is the electric field intensity and Φ_B is the magnetic flux passing through the surface S enclosed by the closed loop C. Since sinusoidal excitation is employed, the magnetic flux density generated by the current also follows a sinusoidal pattern, expressed as $\mathbf{B}(t)=\mathbf{B}_0 e^{j\omega t}$, where $\omega=2\pi f$ is the angular frequency and j is the imaginary unit. The magnetic flux Φ_B is defined as the integral of the magnetic flux density over the area. Given that the geometric structure under consideration remains stationary, the right-hand side of Equation (S1) can be obtained by differentiating the magnetic flux density with respect to time before integrating over the area, yielding:

$$\frac{d\Phi_B}{dt} = j\omega\Phi_B = j(2\pi f)\Phi_B \quad \text{(S2)}$$

Considering a sufficiently small circular sensing region with a characteristic length r, the integral on the left-hand side of Equation (S1) can be evaluated as:

$$\oint_C \mathbf{E} \cdot d\mathbf{l} \approx E \cdot (2\pi r) \quad \text{(S3)}$$

Substituting Equations (S2) and (S3) into Equation (S1) yields $E \approx \frac{2\pi f\Phi_B}{2\pi r}$. Since the amplitude of the excitation current is fixed at 1 pA, the amplitude of the magnetic flux remains constant as well. It can be observed that $E \propto f$, indicating that the initial intensity of the induced electric field is directly proportional to the signal frequency.

Section S2. Calculation of characteristic impedance of different structural segments

The characteristic impedances of different structural segments under high-frequency excitation are calculated based on transmission line theory. As explained in the main text, a first-order lossless transmission line model is adopted for our estimations. For a coaxial transmission line structure, the characteristic impedance is given by:

$$Z=\frac{1}{2\pi}\sqrt{\frac{\mu_0}{\epsilon_0\epsilon_{rm}}}\ln\left(\frac{D}{\mathrm{d}}\right)\approx\frac{60}{\sqrt{\epsilon_{rm}}}\ln\left(\frac{D}{d}\right) \tag{S4}$$

where Z is the characteristic impedance of the structure, μ_0 is the vacuum permeability, and ϵ_{rm} is the relative permittivity of the dielectric insulation layer in the coaxial line. D represents the inner diameter of the transmission line's outer conductor (inclusive of the insulation layer), while d denotes the diameter of the inner conductor. By substituting the corresponding parameters into Equation (S4), the characteristic impedance of the node of Ranvier segment is determined to be $Z_{\mathrm{Ranvier}}\approx 0.282\ \Omega$, and the characteristic impedance of the subsequent myelin sheath structure is found to be $Z_{sheath}\approx 21.67\ \Omega$.

Section S3. Calculation of membrane capacitance and equivalent inductance at the node of Ranvier

The membrane capacitance and equivalent inductance at the node of Ranvier were calculated under idealized conditions. The membrane capacitance at the node of Ranvier is determined by its geometric structure. Since the thickness of the cell membrane is significantly smaller than its circumference, it can be approximated using the parallel-plate capacitor formula: $C=\frac{\varepsilon_r\varepsilon_0 A}{d_{polar}}$, where C is the capacitance, A is the plate area, and d_{polar} is the distance between the plates. By substituting the relevant parameters, the membrane capacitance is calculated as $C_{membrane}$=33.4 fF.

The input impedance formula for the transmission line is given by (with symbol definitions provided in the main text):

$$Z_{in}=Z_{\text{Ranvier}}\frac{Z_{sheath}+jZ_{\text{Ranvier}}\tan(\beta l)}{Z_{\text{Ranvier}}+jZ_{sheath}\tan(\beta l)} \tag{S5}$$

By substituting the existing parameters into the calculation, the input impedance of the subsequent myelin sheath structure is approximately 0.0043-j0.115Ω for a node of Ranvier length of 2 μm. To achieve maximum power transfer efficiency, the output impedance of the sodium ion channels (the signal source) should be 0.0043+j0.115Ω, which is the complex conjugate of the input impedance. The physical relationship between inductive reactance X_L and inductance L is given by:

$$X_L=\omega L=2\pi fL \tag{S6}$$

By substituting the imaginary part of the calculated ideal output impedance of the sodium ion channel into Equation (S6), the equivalent inductance is determined to be $L_{s,eq}$=0.915 fH.

Table S1. Verification of the membrane capacitance and equivalent inductance calculations

Quantity	Calculation	Calculated value
Membrane area, A	$A = \pi dl = \pi(3\ \mu\text{m})(2\ \mu\text{m})$	$1.885 \times 10^{-11}\ \text{m}^2$
Membrane capacitance, $C_{membrane}$	$C = \varepsilon_r\varepsilon_0 A/d_{\text{polar}}$, where $\varepsilon_r = 2$ and $d_{\text{polar}} = 10\ \text{nm}$	33.4 fF
Equivalent inductance, L_{eq}	$L_{\text{s,eq}} = X_L/(2\pi f)$, where $X_L = 0.115\ \Omega$ and $f = 20\ \text{THz}$	0.915 fH
Resonant frequency, f_0	$f_0 = 1/(2\pi\sqrt{L_{\text{s,eq}}C_{\text{membrane}}})$	28.8 THz

Table S2. Simulated initial signal amplitude as a function of nodal length under different axonal geometries at 20 THz

Length of the Node of Ranvier(μm)	Electric Field Strength(μV/m)		
	Baseline (d=3 μm, M=1 μm)	d=5 μm (M = 1 μm)	M=0.5 μm (d = 3 μm)
1	60	120	64
1.25	50	75	57
1.5	77	81	64
1.75	62	78	49
2	**140**	**160**	**93**
2.25	110	140	90
2.5	96	130	88
